# Education Research: Appraisal of Outpatient Clinical Experience During Neurology Residency

**DOI:** 10.1212/NE9.0000000000200046

**Published:** 2023-01-23

**Authors:** Aman Dabir, Vincent Arnone, Beebarg Raza, Umer Najib, Gauri V. Pawar

**Affiliations:** From the Department of Neurology (A.D., V.A., U.N., G.V.P.), Rockefeller Neuroscience Institute, West Virginia University, Morgantown; and Department of Internal Medicine (B.R.), St. Mary Mercy Hospital, Livonia, MI.

## Abstract

**Background and Objectives:**

Outpatient clinical experience is a key component of neurology residency. Understanding the educational environment for residents in the outpatient setting can inform educators to maximize teaching and learning opportunities, enhance resident exposure to subspecialty diagnoses and management, and deliver quality care. We studied the continuity clinic experience of 5 neurology residents over the course of their residency to determine the breadth of their ambulatory experience.

**Methods:**

We used administrative health data from new and return patient visits scheduled with 5 neurology residents of the same class over 3 years of continuity clinic. International classification of disease codes pertaining to neurologic diagnoses and symptoms associated with these visits were analyzed. Frequency and proportions of the most commonly evaluated diagnoses and symptoms were tabulated. These were compared with previously published data about resident experience during training. We also analyzed resident experience over time.

**Results:**

Five neurology residents evaluated 948 patients (mean 189.6; range 180–202; 59.2% female) during 2,699 clinic visits (mean 539.8; range 510–576) over 3 years in their continuity clinics. There were 6,555 international classification of disease codes associated with these visits (2,948 [44.9%] neurologic diagnoses, 2,249 [34.3%] neurologic symptoms, and 1,358 [20.8%] comorbidities). The most common neurologic diagnoses were as follows: headache disorders (24.5%), neuromuscular disorders (17.3%), movement disorders (12.1%), cerebrovascular disorders (11.5%), and epilepsy (7.5%). The most common neurologic symptoms evaluated by residents were as follows: seizure-like events (16.5%), sensory symptoms (12.4%), pain (10.3%), headache (9.7%), and motor symptoms (8.1%).

**Discussion:**

The clinical experience of residents in the continuity clinic was diverse, but it was skewed toward headache, neuromuscular, and movement disorders, which constituted 54% of the workload. When compared with previous studies, the range of resident's outpatient clinical experience differed from that of inpatient experience. Based on the results of this study, we made changes to our outpatient curriculum by adding 2-month–long rotations in subspecialty clinics from postgraduate year 2 to 4 with the aim of boosting resident exposure to neurologic disorders in the outpatient setting.

Postgraduate neurology training takes place in the inpatient, outpatient, and emergency care settings; however, residents spend most time in the inpatient setting.^1^ The relative educational value of training in one setting vs the other has not been evaluated.^2^ It is a commonly held notion that neurologic disorders encountered in the inpatient and outpatient settings are different from each other; however, there have been no direct comparisons. The few studies that assessed trainee experience during residency have shown that cerebrovascular disorders and epilepsy are the 2 most common neurologic disorders evaluated by residents in the inpatient setting.^3,4^ We studied the neurologic diagnoses and symptoms evaluated by 5 neurology residents in their continuity clinics over 3 years of neurology residency to understand the breadth of their outpatient experience. These data would guide educators in maximizing learning opportunities for residents in the outpatient setting. We described the number and type of neurologic diagnoses encountered in the outpatient setting and its change over the course of residency. We compared outpatient and inpatient clinical experience to explore similarities and differences between the 2 of them.

## Methods

### Structure of the Continuity Clinic

During this study (2015–2018), West Virginia University's neurology program had a complement of 20 residents (5 per year). In line with the Accreditation Council for Graduate Medical Education (ACGME) guidelines,^1^ a weekly, half-day continuity clinic was scheduled for each of the 15 residents from postgraduate year (PGY)–2–PGY-4. Eight residents had continuity clinic on Tuesday afternoon, and the rest on Thursday afternoon. Specific faculty members were designated to supervise the continuity clinics. Time spent by the faculty in the continuity clinic represented 10% of their clinical duties. The supervising faculty were subspecialists in headache medicine, neuromuscular medicine, vascular neurology, and epilepsy. Two additional faculty members were available to cross-cover if designated faculty were unavailable. The supervising faculty-to-resident ratio was maintained at 1:3 for every clinic. New patient visits (NPVs) were scheduled for 60 minutes and return patient visits (RPVs) for 30 minutes at all levels of training. Two NPVs and 3 RPVs were scheduled during each 3.5-hour continuity clinic. The clinic was cancelled for a resident when they were on night float rotation, on vacation, attending a national conference, and on hospital-designated holidays. In addition, for PGY-4 residents, continuity clinic was cancelled when they were on inpatient neurology services. To compensate for these cancelled clinics, 6 half-day clinics were added for each resident during their subspecialty clinic rotations. Therefore, each resident had 47, 50, and 47 half-day continuity clinics during PGY-2, PGY-3, and PGY-4, respectively. The neurology clinic is located on the health sciences campus of the West Virginia University in Morgantown, WV. The catchment area of the clinic consists of the entire state of West Virginia along with southwest Pennsylvania, western Maryland, and the upper Ohio valley.

### Data Acquisition and Analysis

We queried the electronic medical record to obtain a list of NPVs and RPVs scheduled with 5 neurology residents of the same class between July 2015 and June 2018. We selected these 5 residents from a cohort of 20 residents—the full complement of residents in the program during the study. The visits represented all continuity clinic patient encounters over 3 years of adult neurology residency training (PGY-2 to PGY-4) for these 5 residents. The following variables were collected for each clinic visit: visit type (new or return), year of visit, patient gender, patient age, associated *International Classification of Disease, Ninth and Tenth Revisions—Clinical Modification* (*ICD-9-CM* and *ICD-10-CM*) disease codes. *ICD-10* codes were exclusively used after January 1, 2016.

Using the *ICD-9* and *ICD-10*, one of the authors (A.D.) obtained disease entries corresponding to each code. To facilitate meaningful analysis, each entry was initially categorized as a neurologic diagnosis, a neurologic symptom, or a comorbidity. Neurologic diagnoses and symptoms were the focus of this study and they were further classified. A neurologic diagnosis was placed in one of these 9 groups: cerebrovascular disorders, cognitive and behavioral disorders, demyelinating disorders, epilepsy, headache disorders, movement disorders, neuromuscular disorders, neuro-oncological disorders, and other neurologic disorders (congenital and structural disorders of the CNS, cranial nerve disorders, encephalopathy, injuries of the CNS, neurologic infections, psychiatric disorders, sleep disorders, spinal cord disorders, and miscellaneous neurologic disorders). These groups were based on the ACGME milestones project implemented during this study.^5^ A neurologic symptom was placed in one of these 11 groups: dizziness, headache, gait abnormality and falls, memory and cognitive symptoms, motor symptoms, pain (including back pain), sensory symptoms, seizure-like events and convulsions, tremor and involuntary movements, visual symptoms, and other neurologic symptoms (abnormal deep tendon reflexes, bladder and bowel symptoms, circadian symptoms, malaise and fatigue, psychiatric symptoms, sequelae of cerebrovascular disease, speech and cranial nerve symptoms, syncope, and miscellaneous symptoms). We also assessed resident experience over the course of their residency to determine trends over time. Deidentified data for the study was available by calendar year (and not by academic year or calendar month). First, we analyzed neurologic diagnoses by calendar year. In a second analysis for trends over time, we divided the 36-month-long continuity clinic experience into two 18-month-long epochs; “the first half” comprising PGY-2 and first 6 months of PGY-3 (July 2015–December 2016), and “the second half” comprising last 6 months of PGY-3 and PGY-4 (January 2017–June 2018). Discrete data were reported as frequencies and proportions (percentage). Epic Electronic Medical Record system (Epic Systems Corporation, Verona, WI) was used by all residents through the duration of this study.

### Standard Protocol Approvals, Registrations, and Patient Consents

The institutional review board of West Virginia University approved the study. Participant consent requirement was waived for this retrospective study.

### Data Availability

Anonymized data not published within this article will be made available by request from any qualified investigator.

## Results

**Figure 1:**
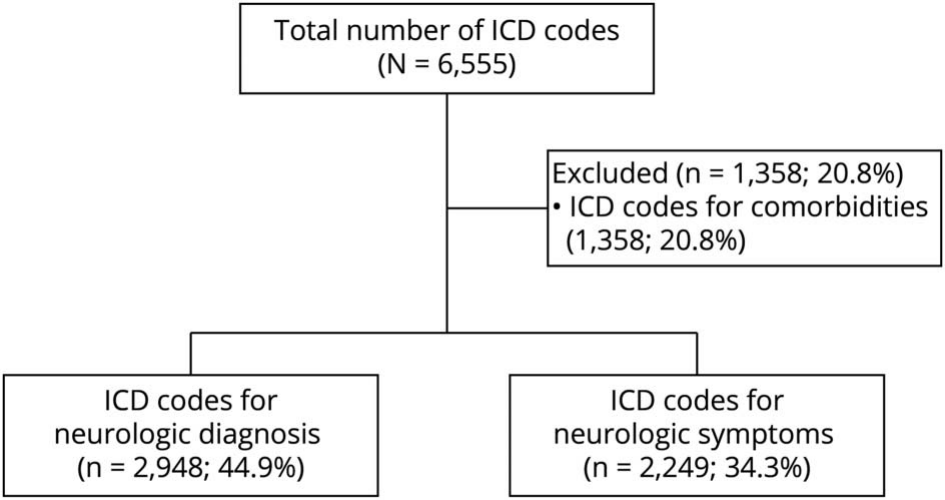
Distribution of *International Classification of Disease* (*ICD*) Codes in 3 Major Categories

The cohort of 5 residents evaluated 948 patients (mean 189.6; range 180–202) from PGY-2 to PGY-4 in their continuity clinics. Of these patients, 562 (59.2%) were female, and the mean age was 50.6 years (range 18–90). Patients were evaluated during 2,699 clinic visits (mean 539.8; range 510–576) of which 1,939 (71.8%) were RPVs and 760 (28.2%) were NPVs. The number of NPVs and RPVs did not differ between the 5 residents (*p* = 0.17; χ^2^ statistic). The “no-show rate” ranged between 9% and 13% for the cohort. There were 6,555 *ICD* codes associated with all patient visits (6,150 *ICD-10* [93.8%] and 405 *ICD-9* [6.2%]). Of these, 2,948 (44.9%) *ICD* codes corresponded to neurologic diagnoses, 2,249 (34.3%) codes corresponded to neurologic symptoms, and the remaining 1,358 (20.8%) codes corresponded to co-morbidities (Figure 1). A patient visit was associated with a mean value of 2.4 *ICD* codes (range 1–7; 1.1 diagnoses, 0.8 symptoms, 0.5 comorbidities).

For the group, headache disorders (24.5%) were the most commonly encountered neurologic disorder. Neuromuscular disorders (17.3%), movement disorders (12.1%), cerebrovascular disorders (11.5%), and epilepsy (7.5%) were the next most common neurologic disorders; see Table 1 for details. Seizure-like events (16.5%) were the most common neurologic symptom evaluated in continuity clinic. This was followed by sensory symptoms (12.4%), pain (10.3%), headache (9.7%), and motor symptoms (8.1%); see Table 2 for details.

**Table 1:** Number (Frequency) of *International Classification of Disease* Codes Pertaining to Neurologic Diagnoses Entered by Each Resident

	Resident 1	Resident 2	Resident 3	Resident 4	Resident 5	Cumulative frequency
Patients evaluated	180	202	189	194	183	948
No. of visits	550	518	545	510	576	2,699
Diagnostic group, frequency (%)						
Headache disorders	137 (21.33)	172 (28.10)	135 (22.57)	116 (32.58)	163 (22.02)	723 (24.53)
Neuromuscular disorders	151 (23.52)	98 (16.01)	113 (18.89)	52 (14.60)	96 (12.97)	510 (17.30)
Movement disorders	95 (14.79)	37 (6.04)	99 (16.55)	41 (11.51)	85 (11.48)	357 (12.11)
Cerebrovascular disorders	55 (8.56)	89 (14.54)	43 (7.19)	53 (14.88)	100 (13.51)	340 (11.53)
Epilepsy	85 (13.23)	32 (5.22)	37 (6.18)	5 (1.40)	63 (8.51)	222 (7.53)
Demyelinating disorders	30 (4.67)	25 (4.08)	24 (4.01)	20 (5.61)	45 (6.08)	144 (4.88)
Cognitive and behavioral disorders	19 (2.95)	34 (5.55)	19 (3.17)	9 (2.52)	30 (4.05)	111 (3.77)
Neuro-oncology	6 (0.93)	7 (1.14)	13 (2.17)	7 (1.96)	9 (1.21)	42 (1.42)
Other diagnoses	64 (9.96)	118 (19.28)	115 (19.23)	53 (14.88)	149 (20.13)	499 (16.93)
Total	642	612	598	356	740	2,948

**Table 2:** Number (Frequency) of *International Classification of Disease* Codes Pertaining to Neurologic Symptoms Entered by Each Resident

	Resident 1	Resident 2	Resident 3	Resident 4	Resident 5	Cumulative frequency
Patients evaluated	180	202	189	194	183	948
No. of visits	550	518	545	510	576	2,699
Symptom group, frequency (%)						
Seizure-like events and convulsions	37 (12.50)	80 (20.83)	114 (20.03)	69 (19.22)	72 (11.23)	372 (16.54)
Sensory symptoms	29 (9.79)	54 (14.06)	55 (9.66)	48 (13.37)	94 (14.66)	280 (12.45)
Pain (including back pain)	33 (11.14)	43 (11.19)	58 (10.19)	32 (8.91)	66 (10.29)	232 (10.32)
Headache	37 (12.50)	59 (15.36)	52 (9.13)	43 (11.97)	29 (4.52)	220 (9.78)
Motor symptoms	28 (9.45)	24 (6.25)	38 (6.67)	31 (8.63)	62 (9.67)	183 (8.14)
Visual symptoms	13 (4.39)	37 (9.63)	47 (8.26)	21 (5.84)	63 (9.82)	181(8.05)
Falls and gait abnormality	33 (11.14)	8 (2.08)	53 (9.31)	16 (4.45)	50 (7.80)	160 (7.11)
Memory and cognitive symptoms	21 (7.09)	10 (2.60)	11 (1.93)	32 (8.91)	49 (7.64)	123 (5.47)
Tremor and involuntary movements	13 (4.39)	8 (2.08)	24 (4.21)	20 (5.57)	22 (3.43)	87 (3.87)
Dizziness	7 (2.36)	14 (3.64)	22 (3.86)	16 (4.45)	19 (2.96)	78 (3.47)
Other symptoms	45 (2.36)	47 (12.23)	95 (16.69)	31 (8.63)	115 (17.94)	333 (14.81)
Total	296	384	569	359	641	2,249

**Figure 2:**
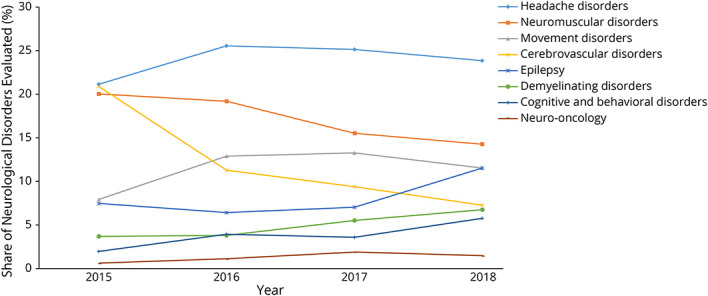
Neurologic Diagnoses Encountered in Continuity Clinic Over the Course of Training 2015–PGY-2 (6 months); 2016–PGY-2 and PGY-3 (6 months each); 2017–PGY-3 and PGY-4 (6 months each); 2018–PGY-4 (6 months). PGY = postgraduate year.

**Figure 3:**
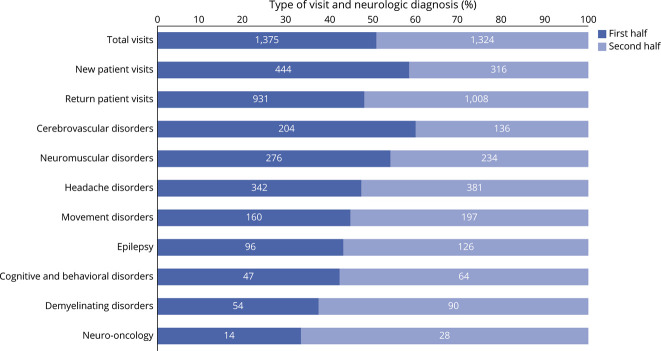
Types of Patient Visits and Neurologic Diagnoses Evaluated During Neurology Residency When Divided Into 2 Halves The first half represents PGY-2 (12 months) and PGY-3 (first 6 months), and the second half represents PGY-3 (last 6 months) and PGY-4 (12 months). PGY = postgraduate year.

During the first-half of continuity clinic experience, 3 residents (60%) entered more diagnosis-related codes than symptom codes. All 5 residents (100%) entered more diagnosis-related codes than symptom codes in the second half of continuity clinic. When compared with the second half, the resident cohort had more NPVs and total patient visits in the first half of continuity clinic training. The case mix evaluated by residents changed over the course of their training: cerebrovascular and neuromuscular disorders were less often encountered by residents as they advanced through training, whereas the proportion of headache, movement demyelinating, cognitive disorders, neuro-oncology, and epilepsy increased (Figures 2 and 3).

## Discussion

In this study of outpatient resident experience during postgraduate training in the United States, a cohort of 5 neurology residents of the same class encountered a variety of neurologic diseases and symptoms in their continuity clinics. However, more than half of the clinical workload was constituted by just 3 neurologic disorders: headache, neuromuscular, and movement disorders. This salient finding is congruent with the natural history of these particular neurologic disorders, which rarely require inpatient management. It is of more interest that the clinical experience of residents in the outpatient setting appears to be noticeably distinct from the inpatient setting. Headache, neuromuscular, and movement disorders, which accounted for 54% of the outpatient diagnosis in our study, made up only 14%–17% of all diagnoses in previously published inpatient focused studies.^3,4^ On the contrary, cerebrovascular disorders and epilepsy, the most commonly encountered diseases evaluated by residents in the inpatient setting, comprised 19% of outpatient diagnoses in our study (Table 3). This contrast of case mix supports the common notion that outpatient and inpatient clinical experiences of neurologists (including trainees) are relatively different. Although certain neurologic disorders such as cerebrovascular disorders and epilepsy are likely to encountered with similar frequency in either setting, others, such as movement, headache, and neuromuscular disorders are near exclusively encountered in the outpatient setting. This finding demonstrates that a resident's outpatient experience is not just a replication of inpatient experience but adds to it in a significant manner. Similar to our results, a survey of 24 outpatient neurology practices in Colorado found neuromuscular and headaches disorders to be the most common neurologic disorder diagnosed in the clinic.^6^ This suggests that the range of outpatient clinical experience during residency mirrors real-life practice of neurology. Over the course of residency, we found that the number of cerebrovascular and neuromuscular disorders encountered by the cohort decreased. However, it increased for all other neurologic disorders. We speculate this may be because the long-term care of these disorders was taken over by primary care providers after they were diagnosed and initially managed in a neurology clinic.

**Table 3:** Comparison of Neurologic Diagnoses Reported in Studies of Neurology Resident Experience in the United States

	Current study West Virginia University (2015–2018)	Moore and Chalk^16^ Mayo Clinic, Rochester (1986–1989)	D'Esposito^3^ Boston University (1988–1991)	Ances^4^ University of Pennsylvania (2002–2005)
Clinical setting	Outpatient (100%)	Inpatient^a^ Outpatient^a^	Inpatient (73%) Outpatient (27%)	Inpatient (75%) Outpatient (25%)
No. of trainees	5	1	1	1
No. of patients evaluated	948	1,009	1,332	1,333
Neurologic disorder (%)				
Headache disorders	24	7	4	6
Neuromuscular disorders	17	20	5	8
Movement disorders	12	2	5	3
Cerebrovascular disorders	11	17	14	18
Epilepsy	7	6	11	12
Others	29	48	61	53

Results rounded off to the nearest integer.

a Data not reported.

Residency training primarily occurs in the inpatient setting, which is in contrast with general neurology practice, which tends to be primarily based in the office setting.^2,6^ Only 6 months of outpatient experience during 36 months of residency is mandated according to current requirements.^1^ The continuity clinic forms the core of this outpatient experience, with a minimum of 40 half-day clinics required per year throughout residency.^1^ Resident and program director surveys have expressed a desire to increase the amount of time spent in the outpatient training,^7-10^ perhaps reflecting the differences between general neurology practice and neurology training. However, there are several challenges in restructuring neurology residency programs, including an increased focus on revenue generation and not teaching, growth of inpatient neurology service lines leading to pressure on time spent in the outpatient setting, and the need for direct supervision of trainees by faculty.^11^ Obtaining robust data that demonstrate the value of outpatient education in neurology education can overcome some of these barriers.

We used administrative health data to gauge resident clinical experience in the outpatient setting. These type of data have been used successfully in the epidemiologic study of diseases,^12^ and they have been validated in the study of neurologic disorders.^13-15^ It allows a thorough assessment of resident's clinical experience by capturing the diagnoses and symptoms evaluated during patient encounters. Unlike previous studies,^3,4,16^ which only analyzed NPVs, we analyzed both new and RPVs to assess the longitudinal experience of residents—the core goal of continuity clinic. Furthermore, we analyzed all neurologic diagnoses associated with a patient visit rather than focusing on the principal diagnosis or chief complaint. It is not uncommon for patients to experience comorbid neurologic disorders; a patient with a remote history of ischemic stroke who develops focal epilepsy and a patient with generalized epilepsy with comorbid migraine. Managing multiple neurologic disorders in a patient contributes to a trainee's experience and should be reflected in any such assessment. To obtain meaningful information from the data set, and when possible, we grouped diagnoses and symptoms into categories. When possible, we grouped neurologic diagnoses according to categories outlined in the ACGME milestones project during the study.^5^ Neurologic symptoms were grouped anatomically or according to sensory domain affected. For example, visual symptoms consisted of *ICD* codes for diplopia, vision loss, decreased vision, and anisocoria. The approach of grouping diagnoses and symptoms may have compensated for the variability among residents in choosing the *ICD* code for a given complaint; for example, a patient reporting tingling and numbness in the feet can be coded as “paresthesia,” “anesthesia,” and “disturbances of skin sensation,” but enumerating it as a sensory symptom overcomes the differences. We assigned specific diagnoses such as migraine, migraine with aura, migraine without aura, and other primary headache disorders (*ICD* codes G43, G44) to the neurologic diagnosis category. However, *ICD* code for headache (R51) was placed in the symptom category. Similarly, we placed epilepsy—focal or generalized (*ICD* G40), in the diagnosis category, whereas unspecified convulsions (R56.9), was placed in the symptom category. We acknowledge that this system was somewhat arbitrary and may have led to overcounting or undercounting diagnoses and symptoms.

This study has at least 6 major limitations. First, we might have overestimated the neurologic diagnoses and symptoms prevalent in the continuity clinic as we enumerated *ICD* codes across multiple visits (NPV and RPV) for a patient. On the contrary, there could be an undercount of diagnoses and symptoms because of the onerous nature of the *ICD* system because residents may not have entered all the pertinent *ICD* codes relevant to an encounter. This may particularly be the case in the early part of residency. Because only 1 *ICD* code is required to close and bill a patient encounter, there is no incentive to enter other diagnosis or symptom codes. Furthermore, the role of supervising attending in choosing *ICD* codes for each encounter was not specifically addressed in our study, and it may have made an impact on the results. Second, this is retrospective study from 1 residency program, and it does not account for interinstitutional differences in disease prevalence, clinic location (urban vs rural), availability of subspecialists in the department, and the presence of other learners (fellows), all of which may affect resident experience. Third, this study evaluated 1 portion of the resident's outpatient experience because in most programs, including ours, residents gain significant skill and knowledge during their subspecialty clinic rotations. Fourth, we did not directly compare the inpatient and outpatient experience of the same set of residents. Fifth, administrative health data have not been previously used in neurology education research. However, it has been validated in the study of other neurologic disorders.^12-15^ Sixth, the sample size of 5 residents evaluated in this study was small. We studied just a single class of residents (from a program complement of 20) to obtain preliminary data as this topic because it has not been studied in great detail in the past. We intended to use these preliminary data to identify areas of improvement in our program and implement changes to our outpatient resident experience. We are currently collecting data prospectively from a larger cohort of residents in our program. Despite these limitations, this study provides first insights into the outpatient experience of neurology trainees during residency and adding to the limited literature on the topic. Unlike previous studies from the United States, which reported single trainee experience from urban academic centers, we report the experience of a cohort of 5 residents of the same class from a program, which primarily serves a rural population.

We found that continuity clinic experience for the residents may have been diverse, but the total number of patients evaluated by each resident over the course of training may not be adequate to prepare them for independent practice. Furthermore, we found that certain neurologic disorders tend to be referred to subspecialty clinics (neuro-oncology, sleep neurology) rather than to the pool of continuity clinic. In response to these findings, we made changes to our program's outpatient curriculum and structure. The major change was introduction of a mandatory outpatient rotation called the subspecialty clinic rotation. During this rotation, residents get an opportunity to evaluate new and return patients while working with various neurology subspecialists for 2 months every year (PGY-2–PGY-4). The schedule for this rotation is made by the program director to ensure relative equity and diversity of clinical exposure. We are currently gathering data from this rotation to determine objectively whether the changes implemented broadened resident's clinical exposure to neurologic disorders in the outpatient setting.

We also made other changes to our program's outpatient training structure based on the results of this preliminary study, which are ongoing. Currently, we are collecting data prospectively from a larger pool of residents in an attempt to improve the quality of data. At the same time, this would allow us to monitor residents' clinical experience as they advance through training. We are also obtaining data from community neurology practices affiliated with our department to compare the disease mix encountered in a general neurology practice with that of our trainees. Please see Table 4 for a summary of changes to our program based on the results of this study.

**Table 4:** Systemic Analysis of Study Findings and Changes Instituted to Our Residency Program

Salient study findings	Residency program changes
The number of patients evaluated by each resident over the course of residency was low and perhaps inadequate to prepare for independent practice	Two subspecialty clinic rotations (1-month long) added each year to the curriculum from PGY-2 to PGY-4 to increase outpatient clinical exposure and supplement continuity clinic experience (implemented)
Lack of exposure to primarily outpatient-based subspecialties such as neuro-oncology, sleep neurology, and neuro-ophthalmology	Residents scheduled to work in these subspecialty clinics during their newly added subspecialty clinic rotation (see above) (implemented)
Differences among residents of the same class in clinical exposure to various neurologic disorders in the outpatient setting	Subspecialty clinic rotation schedule made by the program director to ensure relative parity among trainees; incorporate resident interest in particular subspecialties (implemented)
Differences among residents of the same class in their use of ICD codes	Educate PGY-2 residents about the appropriate use of ICD codes prior to beginning clinic rotations (ongoing)
Inability to compare resident experience during training with Independent practice of general neurology	Obtain data from neurology practices in the community setting affiliated with our department to compare and contrast the 2 (ongoing)
Lack of outpatient data by year of training (PGY-2 to PGY-4) to assess trends over the course of training	Data gathering in prospective manner (ongoing)

Abbreviations: *ICD* = *International Classification of Disease*; PGY = postgraduate year.

There are several knowledge gaps in the subject of resident education and training in the outpatient setting. First, the relative value of inpatient and outpatient training in preparing residents for independent neurology practice is unknown. Future studies comparing resident experience in the inpatient and outpatient settings in the same program during the course of residency may help answer this. Of interest, teaching medical students about neurology in the ambulatory setting compared with inpatient setting was found to be effective.^17^ Second, within the outpatient setting, the educational value of continuity clinics and subspecialty clinics has not been evaluated separately. Third, differences and similarities in clinical experience among residents at the same level of training within and across residency programs has not been evaluated. Our data hint at interresident variability; however, these preliminary findings need to be explored further. We suggest that research studies that collect data prospectively across multiple residency programs can fill some of these knowledge gaps.

Beyond its positive impact on resident experience, educational research in the outpatient clinic offers opportunities for quality improvement. For instance, the ambulatory experience of residents considering fellowships that are primarily based in the outpatient setting (e.g., movement disorders, neuromuscular medicine) can be improvised; thus, these residents evaluate patients with such disorders early in their training (PGY-2), so they can explore their interests well before fellowship applications are due. Another potential quality improvement intervention could be longitudinally monitoring trainee progress in the outpatient setting, including diversity, and extracting the most educational value out of it. Residents who are lagging behind their peers in the evaluation and management of certain neurologic diseases can benefit from more patient encounters pertaining to these disorders. Furthermore, educational research in the outpatient setting can inform decisions about restructuring neurology training such that it is in sync with the requirements of modern neurology practice.

During the course of their residency, 5 neurology residents evaluated a wide range of neurologic disorders in their continuity clinics with headache, neuromuscular, and movement disorders making up half of their workload. This clinical experience appears to be different from previous reports of resident experience in the inpatient setting. We implemented changes to our program's outpatient curriculum based on the results of this study with the aim of improving the quality of residents' outpatient exposure during training and supplementing their continuity clinic experience. There is a need to further evaluate the clinical and educational experience of neurology residents in the outpatient setting.

## Appendix Authors

**Table:**

Name	Location	Contribution
Aman Dabir, MD	Department of Neurology, Rockefeller Neuroscience Institute, West Virginia University, Morgantown	Drafting/revision of the article for content, including medical writing for content; major role in the acquisition of data; study concept or design; and analysis or interpretation of data
Vincent Arnone, MD	Department of Neurology, Rockefeller Neuroscience Institute, West Virginia University, Morgantown	Drafting/revision of the article for content, including medical writing for content; analysis or interpretation of data
Beebarg Raza, MD	Department of Internal Medicine, St. Mary Mercy Hospital, Livonia, MI	Analysis or interpretation of data
Umer Najib, MD	Department of Neurology, Rockefeller Neuroscience Institute, West Virginia University, Morgantown	Drafting/revision of the article for content, including medical writing for content; study concept or design; and analysis or interpretation of data
Gauri V. Pawar, MD	Department of Neurology, Rockefeller Neuroscience Institute, West Virginia University, Morgantown	Drafting/revision of the article for content, including medical writing for content; study concept or design; and analysis or interpretation of data

## Glossary

ACGME: Accreditation Council for Graduate Medical Education
*ICD-9*: *International Classification of Disease, Ninth Revision*
*ICD-10*: *International Classification of Disease, Tenth Revision*
NPV: new patient visit
PGY: postgraduate year
RPV: return patient visit
